# Molecular characteristics of fluoroquinolone-resistant *Escherichia coli* isolated from suckling piglets with colibacillosis

**DOI:** 10.1186/s12866-022-02632-9

**Published:** 2022-09-15

**Authors:** Kwangwon Seo, Kyung-Hyo Do, Wan-Kyu Lee

**Affiliations:** 1 Korea Zoonosis Research Institute, Jeonbuk National University, Iksan, 54531 Republic of Korea; 2 College of Veterinary Medicine, Chungbuk National University, Cheongju, 28644 Korea; 3 Laboratory of Veterinary Bacteriology and Infectious Diseases, College of Veterinary Medicine, Chungbuk National University, Cheongju, 28644 Korea

**Keywords:** *Escherichia coli*, Colibacillosis, Antimicrobial resistance, Fluoroquinolone, Plasmid-mediated quinolone resistance

## Abstract

**Objectives:**

Colibacillosis is a frequent enteric disease in the pig industry that causes significant economic losses. The objective of this study was to investigate the molecular characteristics of fluoroquinolone (FQ)-resistant *E. coli* isolates from suckling piglets with colibacillosis.

**Results:**

A total of 43 FQ-resistant *E. coli* isolates were tested in this study and all isolates showed multi-drug resistance (MDR) and mutations in quinolone resistance determining regions (*gyrA* or *parC*). Especially, FQ-resistant *E. coli* isolates with double mutations in both *gyrA* and *parC* were shown a high FQs minimum inhibitory concentration (≥ 64 mg/L for ciprofloxacin, ≥ 128 mg/L for enrofloxacin, and ≥ 256 mg/L for norfloxacin). Among 43 FQ-resistant *E. coli* isolates, 12 (27.9%) were showed plasmid-mediated quinolone resistance (PMQR) positive *E. coli*. Prevalence of PMQR gene, *aac(6’)-Ib-cr*, *qnrS*, and *qepA*, were identified in 7, 3, and 2 *E. coli* isolates, respectively. We identified the following in PMQR-positive *E. coli* isolates: the tetracycline resistance genes *tetD* (12 isolates, 100.0%), *tetE* (12 isolates, 100.0%), *tetA* (11 isolates, 91.7%), and *tetB* (1 isolate, 8.3%); β-lactamases–encoding *blaCMY-2* (10 isolates, 83.3%), *blaTEM-1* (7 isolates, 58.3%), *blaOXA-1* (7 isolates, 58.3%), *blaSHV-1* (3 isolates, 16.7%), and *blaAAC-2* (1 isolate, 8.3%); and the chloramphenicol resistance genes (10 isolates, 83.3%); the sulfonamide resistance genes *sul1* (9 isolates, 75.0%) and *sul2* (10 isolates, 83.3%); the aminoglycoside modifying enzyme gene *aac(3)-II* (2 isolates, 16.7%). The F4 (7 isolates, 58.3%), LT:STb:EAST1 (5 isolates, 41.7%), and paa (3 isolates, 25.0%) were most common fimbrial antigen, combinations of toxin genes, and non-fimbrial adhesins genes, respectively. All PMQR-positive *E. coli* carried class I integrons but only 4 isolates carried the gene cassette. The most prevalent plasmid replicon was FIB (9 isolates, 75.0%), followed by FIC, HI1, and N (7 isolates, 58.3%), respectively.

**Conclusions:**

Because FQ-resistant *E. coli* can serve as a reservoir of FQ resistant genetic determinants that can be transferred to pathogenic bacteria in humans or pigs, this represents a public health hazard.

**Supplementary Information:**

The online version contains supplementary material available at 10.1186/s12866-022-02632-9.

## Background

Colibacillosis caused by *Escherichia coli* (*E. coli*) in pigs is the most frequent enteric disease and an important cause of death in suckling piglets [1]. This disease may result in significant economic losses in the pig industry because of mortality, decreased weight gain, and costs for treatments, vaccinations and feed supplements [2]. The use of antimicrobial drugs such as β-lactams, aminoglycosides, and fluoroquinolones (FQs) has remained the primary option for controlling colibacillosis.

FQs are broad-spectrum antimicrobials agents and have been used for the treatment of various infections caused by *E. coli* or other gram-negative bacteria. The World Health Organization (WHO) has classified FQs as ‘‘critically important antimicrobials’’ because of their clinical importance in both human and animal medicine [3]. Because the importance of FQs in humans and animals is increasing, FQ-resistant bacteria are a major concern in the treatment of infectious diseases. Notably, FQ-resistant *E. coli* have emerged in Korea after enrofloxacin was licensed to be used for veterinary purposes [4].

Multiple mechanisms are involved in resistance to FQ in Enterobacteriaceae. The major mechanisms of resistance to FQ involve mutations of chromosomal genes encoding DNA gyrase and/or topoisomerase IV [5, 6]. In addition, three different plasmid-mediated quinolone resistance (PMQR) determinants have been described: *qnr*-mediated inhibition of quinolone binding to DNA [7, 8], *qepA* encoded efflux pump [9], and the *aac(6’)-Ib-cr* mediated FQ acetylation [10, 11]. The PMQR genes confer only low-level resistance to FQ; however, they can be spread horizontally among enterobacteria and facilitate the selection of resistant mutants following exposure to FQ [12]. Although studies from several countries have documented the prevalence and characteristics of FQ-resistance in healthy pigs [13–15], there is still limited information regarding the molecular characteristics of FQ-resistant and PMQR-positive *E. coli* isolated from suckling piglets with diarrhea. Therefore, the purpose of this study was to investigate the molecular characteristics of FQ-resistant *E. coli* isolates from suckling piglets with colibacillosis.

## Materials and methods

### Bacterial isolates

*E. coli* isolates collected from each colibacillosis clinical case in suckling piglets from 2007–2018 were tested. The farms consisted of 42 different pig herds (50 to 100 sows per each herd). The aseptically collected intestinal contents and feces were inoculated on MacConkey agar(BD Biosciences, Sparks, MD) containing 4 mg/mL of ciprofloxacin (CIP; Sigma-Aldrich, St.Louis, MO). Subsequently, suspected *E. coli* colonies were identified by VITEK II system (bioMéreiux, France). Thus, a total of 43 CIP-resistant *E. coli* were tested in this study.

### Antimicrobial susceptibility testing

The disk diffusion method was performed according to the Clinical and Laboratory Standards Institute (CLSI) guidelines [16]. The 19 antimicrobial disks (BD Biosciences) used in this study were amikacin (30 μg), amoxicillin-clavulanate (20/10 μg), ampicillin (10 μg), aztreonam (30 μg), cefazolin (30 μg), cephalothin (30 μg), cefoxitin (30 μg), cefepime (30 μg), chloramphenicol (30 μg), colistin (10 μg), doxycycline (30 μg), gentamicin (10 μg), kanamycin (30 μg), nalidixic acid (30 μg), neomycin (30 μg), penicillin (10 U), streptomycin (10 μg), tetracycline (30 μg), and trimethoprim-sulfamethoxazole (1.25/23.75 μg). The minimum inhibitory concentration (MIC) for CIP, enrofloxacin (ENR), and norfloxacin (NOR) was determined by standard agar dilution methods using the Mueller–Hinton agar (BD Biosciences) method according to the guidelines of the CLSI [16]. The breakpoints of CIP, NOR, and ENR were also determined according to the guidelines of the CLSI [16]. *E. coli* ATCC 25,922 was included as a quality control. Multidrug-resistance (MDR) was defined as acquired resistance to at least one agent in three or more antimicrobial classes [17].

### O-Serogroup typing

O-serogroup typing was performed using rabbit antisera purchased from SSI (Serum Staten Institute, Denmark) with the slide agglutination technique of the Animal and Plant Quarantine Agency (Gimcheon, Korea). A standard strain was obtained from Dr. J.M. Fairbrother (OIE *E. coli* Reference Laboratory, Quebec, Canada).

### Identification of mutations in quinolone-resistance-determining regions and detection of plasmid-mediated quinolone-resistance genes

PCR was carried out to amplify quinolone resistance determining regions (QRDRs) of the target genes (*gyrA*, and *parC*) to identify mutations in 43 FQ-resistant *E. coli* isolates using primers and conditions described previously [18, 19]. The PCR products were purified using GFX PCR DNA and the gel band purification kit (Amersham Bioscience, Freiburg, Germany), and sequenced by an automatic sequencer (Cosmogenetech, Seoul, Korea). The sequences were aligned with those in the GenBank nucleotide database using the Basic Local Alignment Search Tool (BLAST) program available through the National Center for Biotechnology Information website (http://www.ncbi.nlm.nih.gov/BLAST). PMQR genes (*qnrA*, *qnrB*, *qnrC*, *qnrD*, *qnrS*, *aac(6’)-Ib-cr*, and *qepA*) were detected by PCR amplification and sequencing analysis, as described in Table S1.

### PCR amplification and sequencing of antimicrobial resistance and virulence genes from PMQR-positive *E. coli*

For detection of antimicrobial resistance genes and virulence genes, PCR was performed using DNA extracted from PMQR-positive *E. coli* isolates. Primers used in the study are listed in Table S1. All PMQR-positive *E. coli* isolates were tested for resistance genes related to aminoglycosides (*aac (3)-II*, and *ant(2’’)-I*), β-lactam antimicrobials (*bla*_*CTX*-M_ families, *bla*_*TEM*_, *bla*_*SHV*_, *bla*_*OXA*_, and pAmpC), chloramphenicols (*cmlA* and *catA1*), sulfonamides (*sul1* and *sul2*), and tetracyclines (*tetA*, *tetB*, *tetC*, *tetD*, *tetE* and *tetG*). The virulence factor genes associated with the toxins (LT, STa, STb, Stx2e, and EAST-1), fimbriae (F4, F5, F6, F18, and F41), and non-fimbrial adhesins (AIDA-1, paa, eae) were also confirmed by PCR as previously described [20].

### Plasmid replicon typing and detection of integrons and gene cassettes

For plasmid replicon typing and detection of integrons and gene cassettes, PCR was performed using DNA extracted from PMQR-positive *E. coli* isolates. The primers used in this study targeted 18 different replicons [21] and class 1 and 2 integrons (Table S1.). Gene cassettes were tested for integron-positive isolates. The PCR products of the gene cassettes were sequenced as described above.

### Conjugation assay

To determine the transferability of PMQR and other genes, conjugation assays were performed using the broth mating method, with *E. coli* J53 used as the recipient as previously described [22]. Transconjugants were selected on MacConkey agar (BD Biosciences) plates containing sodium azide (100 µg/ml; Sigma-Aldrich) and ampicillin or tetracycline (100 μg/ml; Sigma-Aldrich). Transferability was confirmed by antimicrobial susceptibility tests and PCR for molecular analysis as described above.

## Results

### Antimicrobial resistance

MDR patterns of FQ-resistant *E. coli* isolates from colibacillosis are shown in Table 1. Among the 43 FQ-resistant *E. coli*, all isolates showed MDR against 6 to 11 classes of antimicrobial agents. The rates of resistance to the various antimicrobial classes were as follows: aminoglycosides (43/43, 100.0%), penicillins (43/43, 100.0%), quinolones (43/43, 100.0%), tetracyclines (42/43, 97.7%), phenicols (39/43, 90.7%), β-lactam/β-lactamase inhibitor combinations (32/43, 74.4%), folate pathway inhibitors (32/43, 74.4%), cephems (31/43, 72.1%), monobactams (7/43, 16.3%), and polypeptides (6/43, 14.0%). The rate of resistance to 9 antimicrobial classes was the highest at 39.5%, and one (2.3%) FQ-resistant *E. coli* isolate was identified resistance to 11 antimicrobial classes.

**Table 1:** Distribution of multi-drug resistance patterns among 43 fluoroquinolone-resistance *E. coli* isolates

No. of classes	Antimicrobial resistance class pattern	Frequency	Prevalence (%)
11	AMGs, BL/BLICs, CEPs, FQs, FPIs, MONs, PCNs, PHs, PPs, Qs, TETs	1	2.3
10	AMGs, BL/BLICs, CEPs, FQs, FPIs, MONs, PCNs, PHs, Qs, TETs	4	9.3
	AMGs, BL/BLICs, CEPs, FQs, FPIs, PCNs, PHs, PPs, Qs, TETs	3	7.0
9	AMGs, BL/BLICs, CEPs, FQs, FPIs, PCNs, PHs, Qs, TETs	16	37.2
	AMGs, BL/BLICs, FQs, FPIs, MONs, PCNs, PHs, Qs, TETs	1	2.3
8	AMGs, BL/BLICs, FQs, FPIs, PCNs, PHs, Qs, TETs	4	9.3
	AMGs, BL/BLICs, CEPs, FQs, FPIs, PCNs, Qs, TETs	2	4.7
	AMGs, CEPs, FQs, PCNs, PHs, PPs, Qs, TETs	1	2.3
	AMGs, BL/BLICs, CEPs, FQs, FPIs, PCNs, PHs, Qs	1	2.3
7	AMGs, CEPs, FQs, MONs, PCNs, Qs, TETs	1	2.3
	AMGs, CEPs, FQs, PCNs, PHs, Qs, TETs	1	2.3
	AMGs, FQs, PCNs, PHs, PPs, Qs, TETs	1	2.3
6	AMGs, FQs, PCNs, PHs, Qs, TETs	6	14.0
	AMGs, CEPs, FQs, PCNs, Qs, TETs	1	2.3
	Total	43	100.0

*AMGs* Aminoglycosides, *BL/BLICs* β-lactam/β-lactamase inhibitor combinations, *CEPs*, Cephems, *FQs* Fluoroquinolones, *FPIs* Folate pathway inhibitors, *MONs* Monobactams, *PCNs* Penicillins, *PHs*, Phenicols, *PPs* Polypeptides, *Qs* Quionolones, *TETs* Tetracyclines

### Molecular characteristics of FQ-resistant *E. coli*

The molecular characteristics of 43 FQ-resistant *E. coli* isolates are shown in Table 2. Among the isolates, 36 isolates were classified into 17 O-serogroups, and 6 isolates were ungrouped. The most common serogroup was O149 (12 isolates, 27.9%). The *gyrA* amino acid substitutions were S83L (43 isolates), D87N (22 isolates), D87G (5 isolates), and D87E (2 isolates), and the *parC* mutations were S80I (23 isolates), S80R (9 isolates), E84A (5 isolates), S80K (3 isolates), S80N (1 isolates), S80W (1 isolates), E84G (1 isolates), A56C (1 isolates), and S57Q (1 isolates). The MIC ranges for CIP, ENR, and NOR were 4–256 mg/mL, 8–512 mg/ml, and 8–512 mg/mL, respectively, and isolates with double mutations in *gyrA* were relatively higher than those of other isolates with single mutations in *gyrA*. In particular, FQ-resistant *E. coli* isolates with a high level of MICs range (≥ 64 mg/L for CIP, ≥ 128 mg/L for ENR, and ≥ 256 mg/L for NOR) were shown to carry double mutations in *gyrA* in combination with double mutations in *parC*. PMQR genes were detected in 12 (27.9%) of the 43 FQ-resistant *E. coli* isolates. The *aac(6’)-Ib-cr*, *qnrS*, and *qepA* genes were identified in seven, three, and two FQ-resistant *E. coli* isolates, respectively. Among 12 PMQR-positive *E. coli* isolates, one isolate that showed the highest MICs for CIP (256 mg/mL), ENR (512 mg/mL), and NOR (512 mg/mL), also carried the PMQR gene *aac(6’)-Ib-cr*.

**Table 2:** Amino acid changes in the QRDRs, MICs and PMQR determinants of 43 fluoroquinolone-resistance *E. coli* isolates

O Serogroup^a^	PMQR genes^b^	QRDR mutations			MICs (mg/mL)		
		*gyrA*	*parC*		CIP	ENR	NOR
O2	*qnrS*	S83L	WT		4	8	8
O8	-	S83L/D87E	WT		8	16	16
O8	-	S83L/D87N	S80I		16	32	32
O9	-	S83L	S80W		16	64	32
O11	-	S83L/D87N	S80I/E84A		64	256	256
O11	-	S83L/D87N	S80I		16	64	256
O14	*qnrS*	S83L	WT		8	16	16
O27	-	S83L/D87N	S80I		16	64	256
O27	*qepA*	S83L	S80N		16	64	32
O78	-	S83L, D87N	S80I		8	16	32
O101	-	S83L, D87N	S80I		8	16	16
O101	-	S83L, D87N	S80I		16	64	128
O101	-	S83L, D87N	S80I, E84A		64	128	256
O116	-	S83L	S80I		4	16	8
O119	-	S83L	S80I		8	32	32
O127	-	S83L, D87N	S80I, E84A		64	256	256
O127	-	S83L, D87N	S80I		16	64	32
O127	*aac(6’)-Ib-cr*	S83L, D87N	S80I		32	128	256
O147	-	S83L, D87N	S80K		16	32	32
O149	*aac(6’)-Ib-cr*	S83L	A56C, S57Q, S80R		32	64	64
O149	-	S83L, D87G	S80K		8	32	16
O149	*aac(6’)-Ib-cr*	S83L, D87E	WT		16	32	32
O149	-	S83L	E84G		16	64	128
O149	*aac(6’)-Ib-cr*	S83L	S80R		8	16	32
O149	*aac(6’)-Ib-cr*	S83L	S80R		8	32	32
O149	-	S83L, D87N	S80I		16	32	32
O149	-	S83L	S80R		8	32	16
O149	*aac(6’)-Ib-cr*	S83L, D87N	S80I, E84A		256	512	512
O149	*qnrS*	S83L	S80R		4	16	16
O149	-	S83L	S80R		4	16	8
O149	-	S83L, D87N	S80I		8	16	8
O157	-	S83L, D87N	S80K		8	16	16
O159	*qepA*	S83L, D87N	S80I		16	128	256
O167	-	S83L, D87N	S80I		16	64	16
O167	-	S83L	S80I		4	8	8
O182	-	S83L, D87G	S80R		8	32	16
ONT	-	S83L, D87N	S80I		16	32	16
ONT	-	S83L, D87N	S80I		16	64	128
ONT	*aac(6’)-Ib-cr*	S83L, D87N	S80I		32	128	256
ONT	-	S83L, D87G	S80I		8	16	16
ONT	-	S83L, D87N	S80R		8	8	16
ONT	-	S83L, D87G	S80R		8	16	16
ONT	-	S83L, D87G	S80I, E84A		64	128	256

*PMQR* Plasmid-mediated quinolone resistance, *QRDR* Quinolone-resistance determining region, *WT* Wild type, *MICs* Minimum inhibitory concentrations, *CIP* Ciprofloxacin, *ENR* Enrofloxacin, *NOR* Norfloxacin

^a^ONT, not detected

^b^-, not detected

### Characterization of PMQR-positive *E. coli*

The prevalence of antimicrobial resistance genes is shown in Table 3. All PMQR-positive *E. coli* isolates carried the following β-lactamase encoding genes: *bla*_*CMY-2*_ (10 isolates, 83.3%), *bla*_*TEM-1*_ (7 isolates, 58.3%), *bla*_*OXA-1*_ (7 isolates, 58.3%), *bla*_*SHV-1*_ (3 isolates, 16.7%), and *bla*_*AAC-2*_ (1 isolate, 8.3%). Tetracycline-resistance genes were detected in all PMQR-positive *E. coli* isolates as follows: *tetD* (12 isolates, 100.0%), *tetE* (12 isolates, 100.0%), *tetA* (11 isolates, 91.7%), and *tetB* (1 isolate, 8.3%). Two types of aminoglycoside-modifying enzyme genes were examined, but *aac(3)-II* was found only in 2 (16.7%) PMQR-positive *E. coli* isolates. The *sul1* and *sul2* sulfonamide-resistance genes were detected in 9 isolates (75.0%) and 10 isolates (83.3%), respectively. The *cmlA* chloramphenicol-resistance gene was found in 10 isolates (83.3%). Distributions of the virotypes are shown in Table 3. Seven (58.3%) and two (16.7%) of the isolates contained fimbrial antigen F4 and F41, respectively. The most prevalent combinations of toxin genes were LT:STb:EAST1 (5 isolates, 41.7%), followed by EAST1 (3 isolates, 25.0%), LT:STa:STb (1 isolates, 8.3%), STa:STb:EAST1 (1 isolates, 8.3%), STa:STb (1 isolates, 8.3%), Sta (1 isolates, 8.3%). The paa and AIDA non-fimbrial adhesins genes were detected in 3 isolates (25.0%) and 2 isolates (16.7%), respectively. Among 12 PMQR-positive FQ-resistant *E. coli* isolates, all isolates were found to have class 1 integrons. Class 1 integrons contained four types of gene cassette arrangements, *aadA1*-*dfrA1* (2 isolates), *aadA1*-*aadA2*- *aadB* (1 isolate), and *aadA1*-*aadA2*- *aadB*-*cmlA6* (1 isolate). Eight isolates did not carry any of the gene cassettes. A total of 10 plasmid replicon types were identified in all 12 PMQR-positive *E. coli* isolates. The most common plasmid replicon was FIB (9 isolates, 75.0%), followed by FIC, HI1, and N (7 isolates, 58.3%), respectively. Transferability was only identified in 7 isolates among 12 PMQR-positive FQ-resistant *E. coli* isolates.

**Table 3:** Phenotypes and genotypes of 12 PMQR-positive *E. coli* isolates

Isolate	PMQR gene	Resistance phenotypes	Resistance genes	Integron and gene cassettes	Plasmid replicon type	Virotype
SSC-8	*qepA*	AM, AMC, C, CF, CL, CZ, D, GM, K, N, NA, P, S, SXT, TE	*bla*_*TEM-1*_, *bla*_*CMY-2*_, *sul1*, *cmlA*, *tetA*, *tetD*, *tetE*	I (*aadA1-dfrA1*)	I1	EAST1
SSC-23	*qepA*	AM, AMC, AN, C, CF, CZ, D, GM, K, N, NA, P, S, SXT, TE	*bla*_*TEM-1*_, *bla*_*CMY-2*_, *sul1*, *sul2*, *cmlA*, *tetA*, *tetD*, *tetE*	I (*aadA1*-*aadA2*- *aadB*-*cmlA6*)	FIB, I1, P	EAST1
SSC-29	*aac(6’)-Ib-cr*	AM, AMC, C, D, GM, K, N, NA, P, S, SXT, TE	*bla*_*TEM-1*_, *bla*_*SHV-1*_, *bla*_*OXA-1*_, *sul1*, *sul2*, *cmlA*, *tetA*, *tetD*, *tetE*	I (-)	FIB, FIC, HI1, N	F4:paa:LT:STb:EAST1
SSC-30	*aac(6’)-Ib-cr*	AM, AMC, C, CF, D, GM, K, N, NA, P, SXT, TE	*bla*_*OXA-1*_, *sul1*, *sul2*, *cmlA*, *aac(3)-II*, *tetA*, *tetD*, *tetE*	I (*aadA1-dfrA1*)	I1, HI1, N, Y	F4:F41:STa:STb:EAST1
SSC-31	*aac(6’)-Ib-cr*	AM, AMC, C, CF, CZ, FEP, FOX, GM, K, N, NA, P, SXT, TE	*bla*_*OXA-1*_, *bla*_*CMY-2*_, *sul1*, *sul2*, *cmlA*, *tetD*, *tetE*	I (-)	FIB, FIC, HI1, N	F4:LT:STb:EAST1
SSC-33	*aac(6’)-Ib-cr*	AM, AMC, C, D, GM, K, N, NA, P, S, SXT, TE	*bla*_*OXA-1*_, *bla*_*CMY-2*_, *sul1*, *sul2*, *cmlA*, *tetA*, *tetD*, *tetE*	I (-)	FIA, FIB, FIC, HI1, N, Y	F4:paa:AIDA:LT:STb:EAST1
SSC-35	*qnrS*	AMC, CF, D, GM, K, N, NA, P, S, TE	*bla*_*CMY-2*_, *sul1*, *tetA*, *tetB*, *tetD*, *tetE*	I (*aadA1*-*aadA2*- *aadB*)	FIB, I1, X	STa
SSC-37	*aac(6’)-Ib-cr*	AM, AMC, C, D, GM, K, N, NA, P, S, SXT, TE	*bla*_*TEM-1*_, *bla*_*SHV-1*_, *bla*_*OXA-1*_, *bla*_*CMY-2*_, *sul2*, *cmlA*, *tetA*, *tetD*, *tetE*	I (-)	FIB, FIC, HI1, N	F4:paa:AIDA:LT:STa:STb
SSC-38	*aac(6’)-Ib-cr*	AM, AMC, C, CF, CZ, D, GM, K, N, NA, P, S, SXT, TE	*bla*_*TEM-1*_, *bla*_*SHV-1*_, *bla*_*OXA-1*_, *bla*_*CMY-2*_, *sul1*, *sul2*, *cmlA*, *tetA*, *tetD*, *tetE*	I (-)	FIB, FIC, HI1, N	EAST1
SSC-41	*aac(6’)-Ib-cr*	AM, AMC, C, CF, D, GM, K, N, NA, P, S, SXT, TE	*bla*_*OXA-1*_, *bla*_*CMY-2*_, *sul1*, *sul2*, *cmlA*, *tetA*, *tetD*, *tetE*	I (-)	FIB, FIC, HI1, N, Y	LT:STb:EAST1
SSC-42	*qnrS*	AM, CF, D, GM, NA, P, S, SXT, TE	*bla*_*TEM-1*_, *bla*_*CMY-2*_, *sul2*, *aac(3)-II*, *tetA*, *tetD*, *tetE*	I (-)	FIA, FIB, FIC, I1, X	F4:LT:STb:EAST1
SSC-47	*qnrS*	AM, AMC, AN, ATM, C, CZ, D, K, N, NA, P, S, SXT, TE	*bla*_*TEM-1*_, *bla*_*ACC-2*_*, bla*_*CMY-2*_, *sul2*, *cmlA*, *tetA*, *tetD*, *tetE*	I (-)	A/C, I1	F4:F41:STa:STb

Underline indicate that was found in the transconjugant strains

*PMQR* Plasmid-mediated quinolone resistance, *AM* Ampicillin, *AMC* Amoxicillin-clavulanic acid, *AN* Amikacin, *ATM* Aztreonam, *C* Chloramphenicol, *CF* Cephalothin, *CL* Colistin, *CZ* Cefazolin, *D* Doxycycline, *FEP*, Cefepime, *FOX* Cefoxitin, *GM* Gentamicin, *K* Kanamycin, *N* Neomycin, *NA* Nalidixic acid, *P* Penicillin, *S* Streptomycin, *SXT* Sulfamethoxazole/trimethoprim, *TE* Tetracycline

## Discussion

Suckling piglets are vulnerable to colibacillosis for many reasons such as changes in environmental conditions, a decline in maternal antibody titers, and various stresses. Antimicrobials are used in intensive pig production systems to control infectious diseases. In particular, FQs are highly effective antimicrobial class with many advantages including high oral absorption, large volume of distribution, and broad-spectrum antimicrobial activity [23]. However, increasing use of these agents has led to rising rates of resistance to FQs in *E. coli* worldwide [24]; thus, the probability of treatment failure may be increased [25]. In this study, all FQ-resistant *E. coli* isolates showed were MDR (resistance to more than six antimicrobial agents) with high levels of resistance to several antimicrobials: aminoglycosides (100.0%), penicillins (100.0%), tetracyclines (97.7%), and phenicols (90.7%). In particular, five isolates showed resistance to more than 10 classes. These results were consistent with previous studies showing co-association of resistance to other classes of antimicrobials and high MDR rates among FQ-resistant *E. coli* [26, 27]. This occurs because PMQR genes and other antimicrobial-resistance genes are linked together on the plasmid [26].

The O-serogroup is considered one of the major virulence factors of *E. coli* and variety of O-serogroups has been associated with diarrhea [28–30]. In the present study, 17 O-serogroups were detected and O149 was most common. The O149 serogroup has been determined to be the dominant serogroup in cases of diarrhea in many countries including China and Canada [31, 32]. In addition, in this study, all isolates (100%) had *gyrA* mutations, and 29 isolates (67.4%) were double amino acid substitutions (S83L plus substitution in aspartic acid 87). These results were consistent with previous studies showing that DNA gyrase is the primary target of FQ in gram-negative bacteria, and *gyrA* mutations are dominant mutations in *E. coli* [33]. Moreover, 37 (86.0%) FQ-resistant *E. coli* isolates had mutations at codon 80 in *parC* in the QRDRs, and the most common type of amino-acid substitution was S80I in *parC*. Previous studies reported that the substitution S80I was most frequently observed among substitutions in the QRDR of *parC* [34, 35]. These results are consistent with those observed in *E. coli* isolates from humans [36, 37]. Also, the MICs ranges of isolates with double mutations in *gyrA* were relatively higher than those of other isolates with single mutations in *gyrA*. Notably, FQ-resistant *E. coli* isolates carrying double mutations in *gyrA* in combination with double mutations in *parC* were identified at high levels of MICs (≥ 64 mg/L for CIP, ≥ 128 mg/L for ENR, and ≥ 256 mg/L for NOR). These results are consistent with those of recent studies showing that the total number of point mutations in QRDR was positively correlated with the increased MIC.

In this study, 12 FQ-resistant *E. coli* isolates carried three types of PMQR genes, *aac(6’)-Ib-cr* (7 isolates), *qnrS* (3 isolates), and *qepA* (2 isolates). These PMQR variants have been previously detected in *E. coli* from livestock, including in healthy animals and retail meats in the Czech Republic [38], China [39], and the United States [40], as well as from swine in Korea [14]. Also, to acquire FQ resistance, bacteria usually require at least double mutations in QRDRs [41]. However, in this study, two isolates (4 mg/L for CIP, 8 mg/L for ENR, and 8 mg/L for NOR) had only a single mutation in *gyrA* and harbored *qnrS* in its plasmid. This result showed that PMQR genes play a role in FQ resistance [42].

The rise of antimicrobial resistance is thought to be closely associated with the widespread transfer of resistance genes between bacterial species. In this study, all 12 PMQR-positive *E. coli* isolates carried a variety of antimicrobial resistance genes such as *blaCMY*, *blaTEM*, *blaOXA*, *blaSHV*, *blaAAC*, *tetA*, *tetB*, *tetD*, *tetE*, *aac(3)-II*, *sul1*, *sul2*, and *cmlA* and harbored mobile elements such as integrons and gene cassettes at the same time. The *bla* genes hydrolyze the characteristic β-lactam ring and confer resistance to most β-lactam antimicrobials, including cephalosporins [43]. Previous studies reported that the PMQR genes in *bla* positive-*E. coli* were detected at high levels [44]. The presence of the PMQR genes may be significantly associated with the β-lactamase gene, perhaps due to common carriage on a plasmid in Enterobacteriaceae [45]. Also, all 12 PMQR-positive *E. coli* isolates harbored class 1 integrons and four isolates also contained gene cassettes *aadA* or *dfrA* or both genes. These genes are frequently detected in class 1 integrons isolated from animals and humans in Korea [46]. Therefore, integrons in PMQR-positive *E. coli* isolates from suckling piglets may have acquired the genetic determinants of drug resistance, which could become a concern.

Plasmids are small DNA molecules that are distinct from chromosomes and can provide beneficial effects to bacteria such as antibiotic resistance through horizontal gene transfer [21]. In this study, 10 plasmid replicon types were identified in all 12 PMQR-positive *E. coli* isolates. The most common plasmid replicon was IncF plasmids including FIB, and FIC. IncF plasmids were associated with the important role in the spread of virulence and resistance to important classes of antimicrobials including quinolones, β-lactams, TEs, sulfonamides, chloramphenicol, and aminoglycosides among Enterobacteriaceae [47].

The detection of *E. coli* virulence factors is important for diagnosing and establishing preventative measures for colibacillosis [48]. In this study, toxin genes LT, STb, and Sta were detected in nine (75.0%), nine (75.0%), and one (8.3%) PMQR-positive *E. coli* isolates, respectively. These LT, STa, and STb genes damage the vessel and cause edema and a high mortality in pigs [49]. Also, the most prevalent fimbriae antigen was F4 (7 isolates, 58.3%). F4 fimbriae have been frequently detected in piglets in several countries such as Japan, Europe, and the United States [50–52]. Previous studies reported that paa is known to have a high association with F4 [50, 53]. In this study, 6 (50.0%) PMQR-positive *E. coli* isolates were identified as having the paa gene and coexisting with the F4 gene. Although the specific role of the paa gene in the development of pathogenic *E. coli* has not yet been clearly defined, various virotypes may also appear as a result of horizontal gene transferability of the paa gene [54].

## Conclusions

This study investigated the molecular characteristics of FQ-resistant *E. coli* isolated from suckling piglets with colibacillosis. All FQ-resistant *E. coli* isolates showed an MDR phenotype, and the most prevalent of the mutations were double point mutations in *gyrA* and a single mutation in *parC*. Also, FQ-resistant *E. coli* isolates with PMQR genes carried various antimicrobial genes and harbored mobile elements and plasmid replicons. Antimicrobial resistance may become a serious problem because many drugs are probably ineffective for the treatment of colibacillosis and resistance elements can be horizontally transferred on pig farms. Also, this represents a public health hazard because FQ-resistant *E. coli* can serve as a reservoir of FQ resistant genetic determinants that can be transferred to pathogenic bacteria in humans or pigs. These data support the critical need for comprehensive surveillance of antimicrobial resistance on pig farms.

## Supplementary Information

**Additional file 1:** **Figure S1-1**. LC716475 (SSC-1) blast alignment. **Figure S1-2**. LC716476 (SSC-2) blast alignment. **Figure S1-3**. LC716477 (SSC-3) blast alignment. **Figure S1-4**. LC716478 (SSC-4) blast alignment. **Figure S1-5**. LC716479 (SSC-7) blast alignment. **Figure S1-6**. LC716480 (SSC-8) blast alignment. **Figure S1-7**. LC716481 (SSC-10) blast alignment. **Figure S1-8**. LC716482 (SSC-11) blast alignment. **Figure S1-9**. LC716483 (SSC-12) blast alignment. **Figure S1-10**. LC716484 (SSC-13) blast alignment. **Figure S1-11**. LC716485 (SSC-14) blast alignment. **Figure S1-12**. LC716486 (SSC-15) blast alignment. **Figure S1-13**. LC716487 (SSC-16) blast alignment. **Figure S1-14**. LC716488 (SSC-17) blast alignment. **Figure S1-15**. LC716489 (SSC-19) blast alignment. **Figure S1-16**. LC716490 (SSC-20) blast alignment. **Figure S1-17**. LC716491 (SSC-21) blast alignment. **Figure S1-18**. LC716492 (SSC-22) blast alignment. **Figure S1-19**. LC716493 (SSC-23) blast alignment. **Figure S1-20**. LC716494 (SSC-26) blast alignment. **Figure S1-21**. LC716495 (SSC-27) blast alignment. **Figure S1-22**. LC716496 (SSC-28) blast alignment. **Figure S1-23**. LC716497 (SSC-29) blast alignment. **Figure S1-24**. LC716498 (SSC-30) blast alignment. **Figure S1-25**. LC716499 (SSC-31) blast alignment. **Figure S1-26**. LC716500 (SSC-32) blast alignment. **Figure S1-27**. LC716501 (SSC-33) blast alignment. **Figure S1-28**. LC716502 (SSC-34) blast alignment. **Figure S1-29**. LC716503 (SSC-35) blast alignment. **Figure S1-30**. LC716504 (SSC-36) blast alignment. **Figure S1-31**. LC716505 (SSC-37) blast alignment. **Figure S1-32**. LC716506 (SSC-38) blast alignment. **Figure S1-33**. LC716507 (SSC-39) blast alignment. **Figure S1-34**. LC716508 (SSC-40) blast alignment. **Figure S1-35**. LC716509 (SSC-41) blast alignment. **Figure S1-36**. LC716510 (SSC-42) blast alignment. **Figure S1-37**. LC716511 (SSC-43) blast alignment. **Figure S1-38**. LC716512 (SSC-44) blast alignment. **Figure S1-39**. LC716513 (SSC-45) blast alignment. **Figure S1-40**. LC716514 (SSC-46) blast alignment. **Figure S1-41**. LC716515 (SSC-47) blast alignment. **Figure S1-42**. LC716516 (SSC-48) blast alignment. **Figure S1-43**. LC716517 (SSC-49) blast alignment.

**Additional file 2:** **Table S1**. Primers used for PCR and DNA sequencing.

**Additional file 3:** **Table S2**. History of samples and isolates separated from each sample.

## Data Availability

Quinolone resistance determining regions sequences of the *E. coli* isolates have been deposited in DDBJ database (http://getentry.ddbj.nig.ac.jp/) under the accession numbers LC716475 (SSC-1), LC716476 (SSC-2), LC716477 (SSC-3), LC716478 (SSC-4), LC716479 (SSC-7), LC716480 (SSC-8), LC716481 (SSC-10), LC716482 (SSC-11), LC716483 (SSC-12), LC716484 (SSC-13), LC716485 (SSC-14), LC716486 (SSC-15), LC716487 (SSC-16), LC716488 (SSC-17), LC716489 (SSC-19), LC716490 (SSC-20), LC716491 (SSC-21), LC716492 (SSC-22), LC716493 (SSC-23), LC716494 (SSC-26), LC716495 (SSC-27), LC716496 (SSC-28), LC716497 (SSC-29), LC716498 (SSC-30), LC716499 (SSC-31), LC716500 (SSC-32), LC716501 (SSC-33), LC716502 (SSC-34), LC716503 (SSC-35), LC716504 (SSC-36), LC716505 (SSC-37), LC716506 (SSC-38), LC716507 (SSC-39), LC716508 (SSC-40), LC716509 (SSC-41), LC716510 (SSC-42), LC716511 (SSC-43), LC716512 (SSC-44), LC716513 (SSC-45), LC716514 (SSC-46), LC716515 (SSC-47), LC716516 (SSC-48), and LC716517 (SSC-49).

## Abbreviations

*E. coli*: *Escherichia coli*
FQ: Fluoroquinolone
CIP: Ciprofloxacin
PMQR: Plasmid-mediated quinolone resistance
MIC: Minimum inhibitory concentration
ENR: Enrofloxacin
NOR: Norfloxacin
MDR: Multidrug-resistance
QRDRs: Quinolone resistance determining regions
