# Supplementary material for: Molecular characteristics of fluoroquinolone-resistant Escherichia coli isolated from suckling piglets with colibacillosis
Source: BMC Microbiol. 2022 Sep 15;22:216. doi: 10.1186/s12866-022-02632-9 (PMC9476276; doi:10.1186/s12866-022-02632-9)
Supplement: Supplementary file 1 — Additional file 1: Figure S1-1. LC716475 (SSC-1) blast alignment. Figure S1-2. LC716476 (SSC-2) blast alignment. Figure S1-3. LC716477 (SSC-3) blast alignment. Figure S1-4. LC716478 (SSC-4) blast alignment. Figure S1-5. LC716479 (SSC-7) blast alignment. Figure S1-6. LC716480 (SSC-8) blast alignment. Figure S1-7. LC716481 (SSC-10) blast alignment. Figure S1-8. LC716482 (SSC-11) blast alignment. Figure S1-9. LC716483 (SSC-12) blast alignment. Figure S1-10. LC716484 (SSC-13) blast alignment. Figure S1-11. LC716485 (SSC-14) blast alignment. Figure S1-12. LC716486 (SSC-15) blast alignment. Figure S1-13. LC716487 (SSC-16) blast alignment. Figure S1-14. LC716488 (SSC-17) blast alignment. Figure S1-15. LC716489 (SSC-19) blast alignment. Figure S1-16. LC716490 (SSC-20) blast alignment. Figure S1-17. LC716491 (SSC-21) blast alignment. Figure S1-18. LC716492 (SSC-22) blast alignment. Figure S1-19. LC716493 (SSC-23) blast alignment. Figure S1-20. LC716494 (SSC-26) blast alignment. Figure S1-21. LC716495 (SSC-27) blast alignment. Figure S1-22. LC716496 (SSC-28) blast alignment. Figure S1-23. LC716497 (SSC-29) blast alignment. Figure S1-24. LC716498 (SSC-30) blast alignment. Figure S1-25. LC716499 (SSC-31) blast alignment. Figure S1-26. LC716500 (SSC-32) blast alignment. Figure S1-27. LC716501 (SSC-33) blast alignment. Figure S1-28. LC716502 (SSC-34) blast alignment. Figure S1-29. LC716503 (SSC-35) blast alignment. Figure S1-30. LC716504 (SSC-36) blast alignment. Figure S1-31. LC716505 (SSC-37) blast alignment. Figure S1-32. LC716506 (SSC-38) blast alignment. Figure S1-33. LC716507 (SSC-39) blast alignment. Figure S1-34. LC716508 (SSC-40) blast alignment. Figure S1-35. LC716509 (SSC-41) blast alignment. Figure S1-36. LC716510 (SSC-42) blast alignment. Figure S1-37. LC716511 (SSC-43) blast alignment. Figure S1-38. LC716512 (SSC-44) blast alignment. Figure S1-39. LC716513 (SSC-45) blast alignment. Figure S1-40. LC716514 (SSC-46) blast alignment. Figure S1-41. LC716515 (SSC-47) blas [file 12866_2022_2632_MOESM1_ESM.docx]

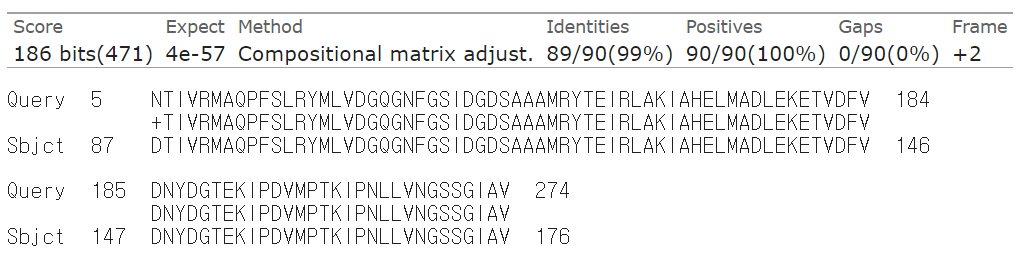


**Figure S1-1.** LC716475 (SSC-1) blast alignment.


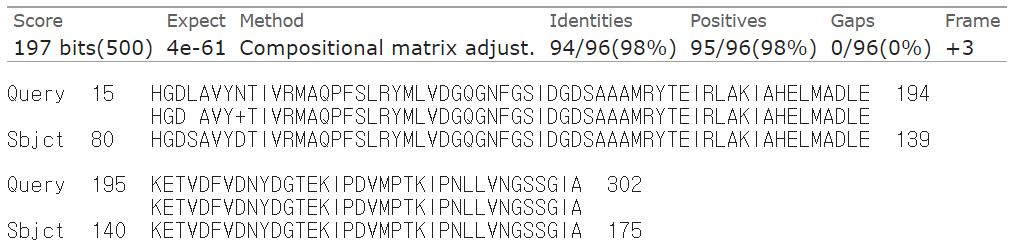


**Figure S1-2.** LC716476 (SSC-2) blast alignment.


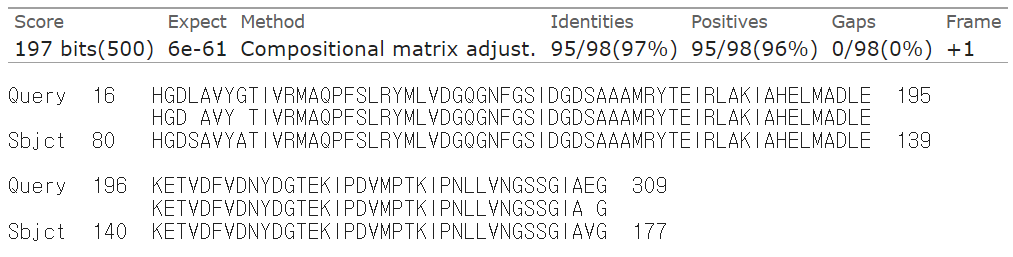


**Figure S1-3.** LC716477 (SSC-3) blast alignment.


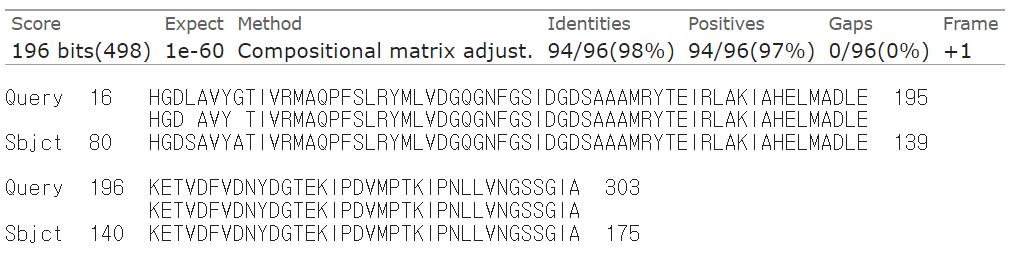


**Figure S1-4.** LC716478 (SSC-4) blast alignment.


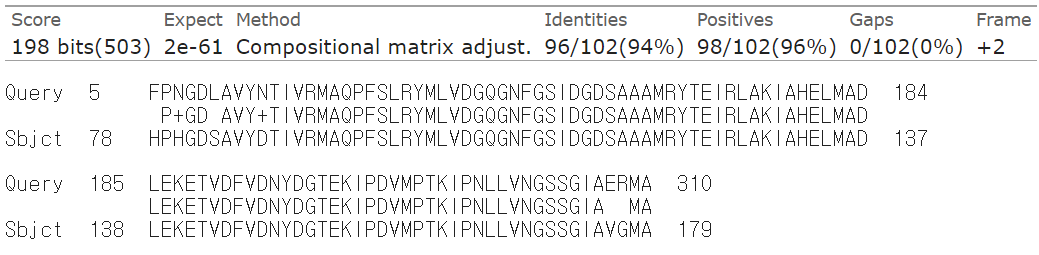


**Figure S1-5.** LC716479 (SSC-7) blast alignment.


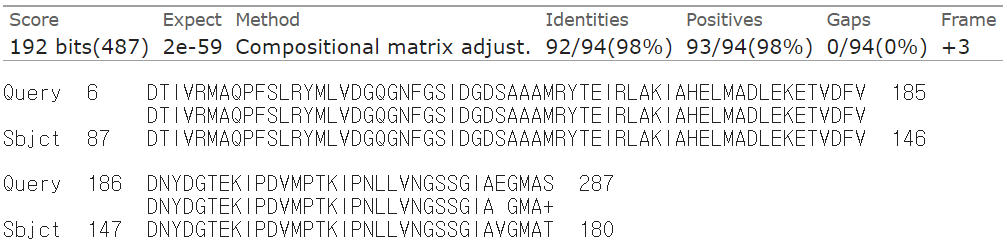


**Figure S1-6.** LC716480 (SSC-8) blast alignment.


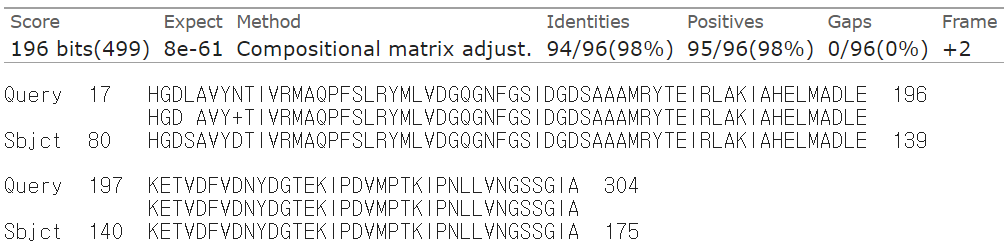


**Figure S1-7.** LC716481 (SSC-10) blast alignment.


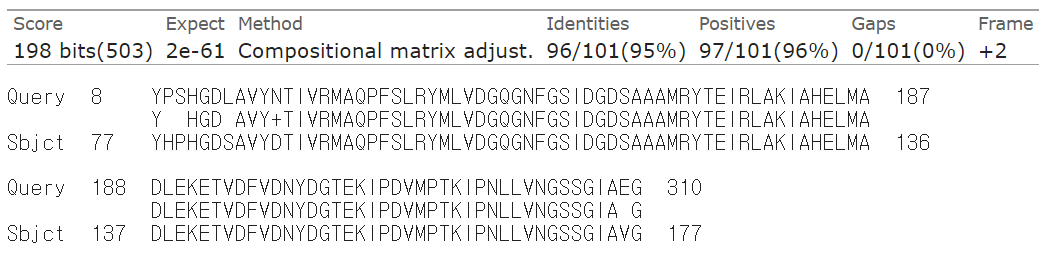


**Figure S1-8.** LC716482 (SSC-11) blast alignment.


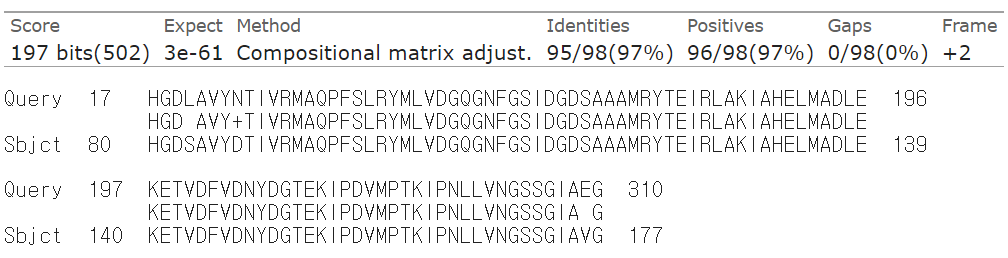


**Figure S1-9.** LC716483 (SSC-12) blast alignment.


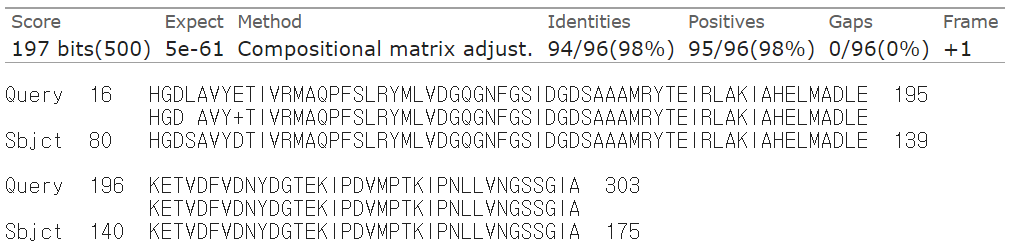


**Figure S1-10.** LC716484 (SSC-13) blast alignment.


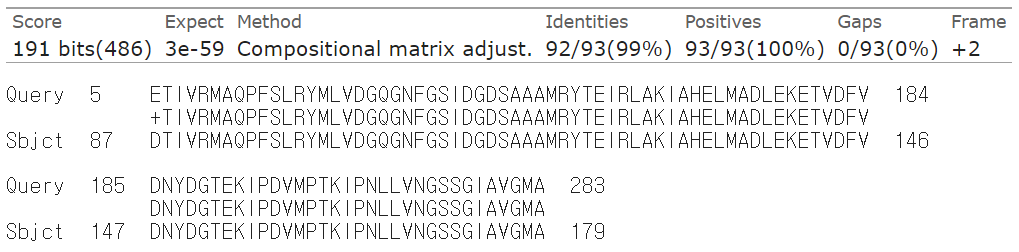


**Figure S1-11.** LC716485 (SSC-14) blast alignment.


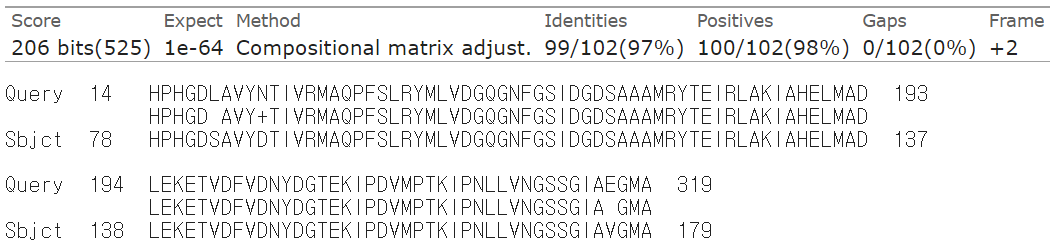


**Figure S1-12.** LC716486 (SSC-15) blast alignment.


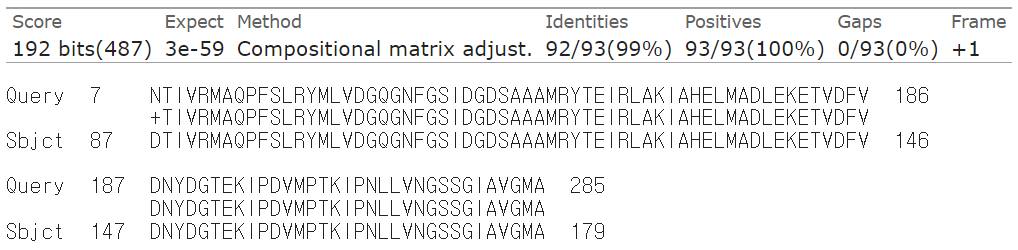


**Figure S1-13.** LC716487 (SSC-16) blast alignment.


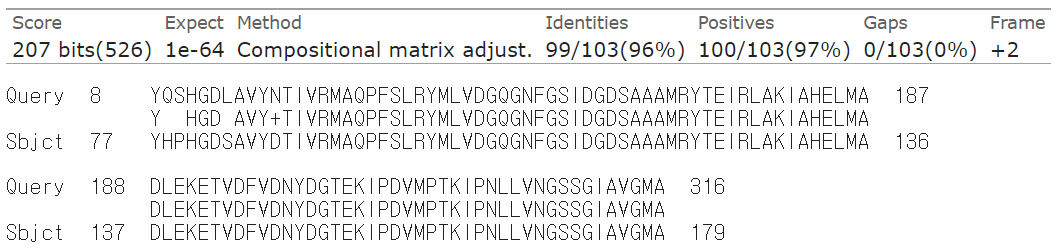


**Figure S1-14.** LC716488 (SSC-17) blast alignment.


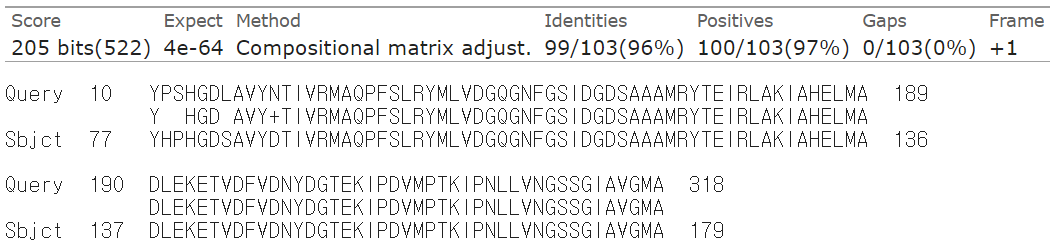


**Figure S1-15.** LC716489 (SSC-19) blast alignment.


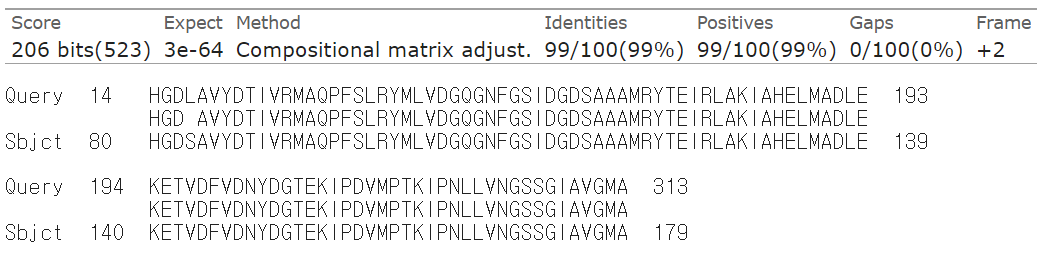


**Figure S1-16.** LC716490 (SSC-20) blast alignment.


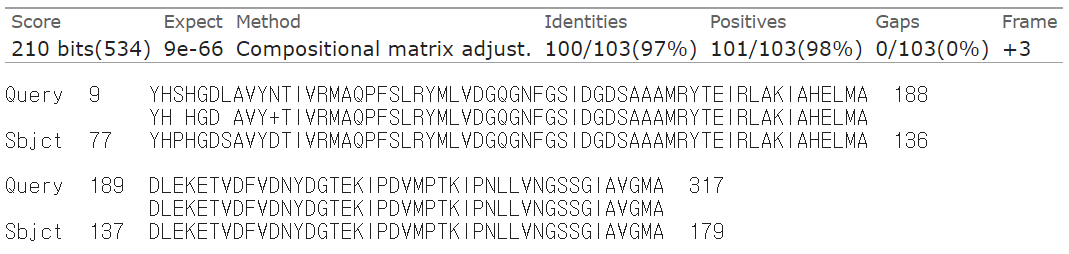


**Figure S1-17.** LC716491 (SSC-21) blast alignment.


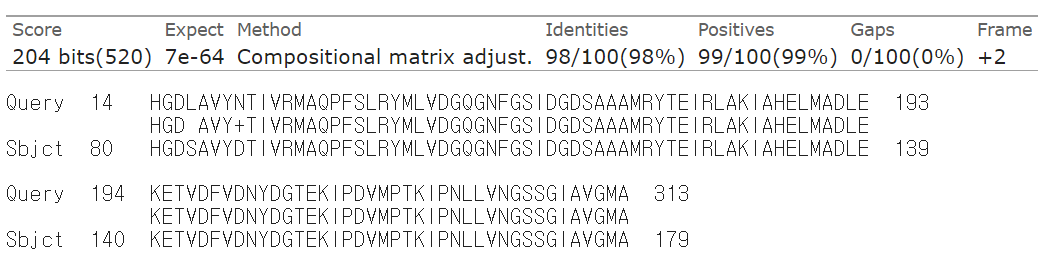


**Figure S1-18.** LC716492 (SSC-22) blast alignment.


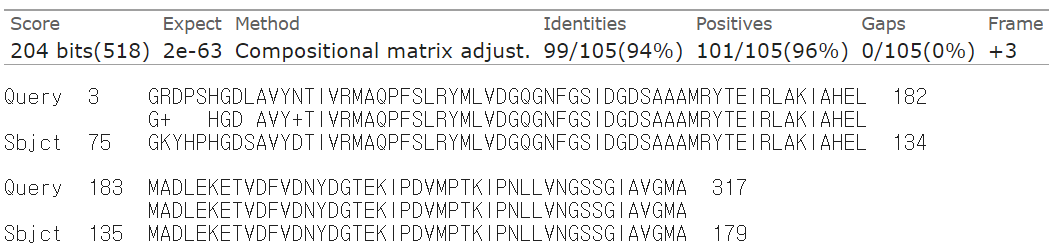


**Figure S1-19.** LC716493 (SSC-23) blast alignment.


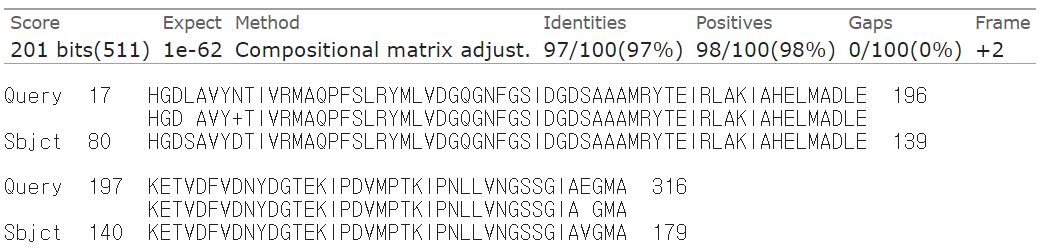


**Figure S1-20.** LC716494 (SSC-26) blast alignment.


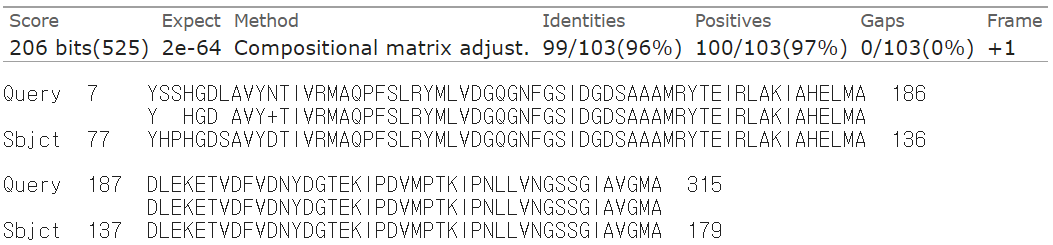


**Figure S1-21.** LC716495 (SSC-27) blast alignment.


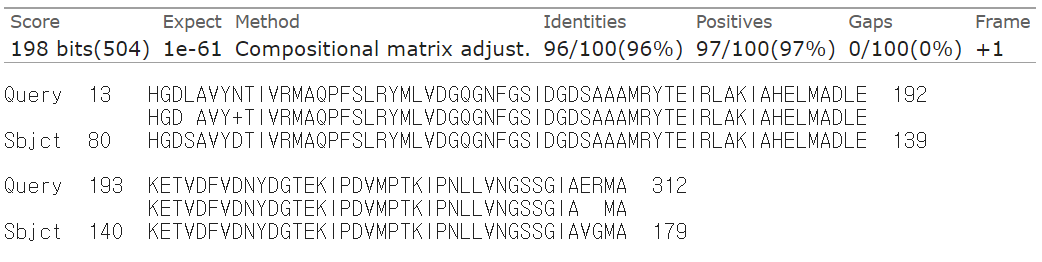


**Figure S1-22.** LC716496 (SSC-28) blast alignment.


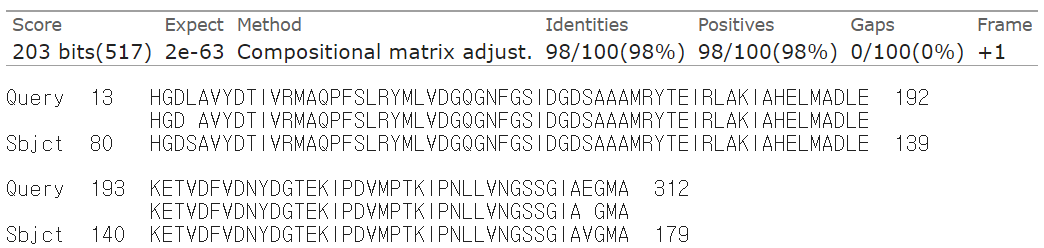


**Figure S1-23.** LC716497 (SSC-29) blast alignment.


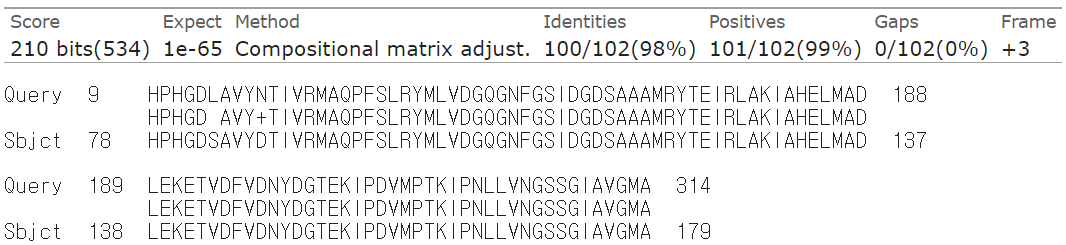


**Figure S1-24.** LC716498 (SSC-30) blast alignment.


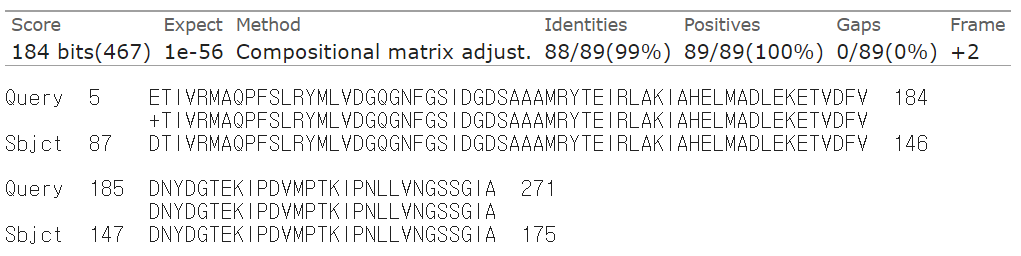


**Figure S1-25.** LC716499 (SSC-31) blast alignment.


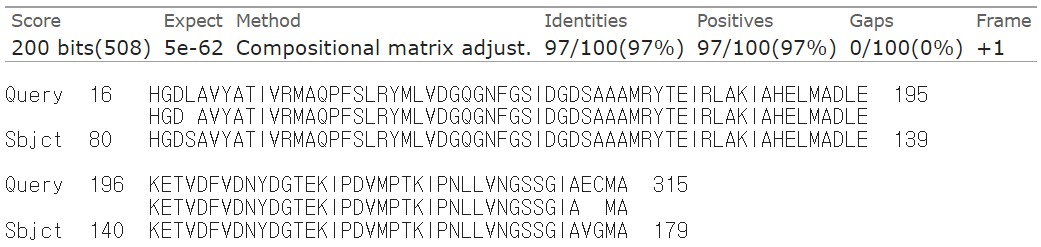


**Figure S1-26.** LC716500 (SSC-32) blast alignment.


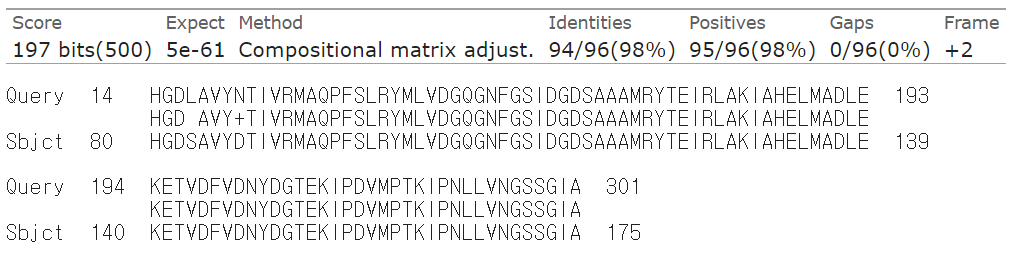


**Figure S1-27.** LC716501 (SSC-33) blast alignment.


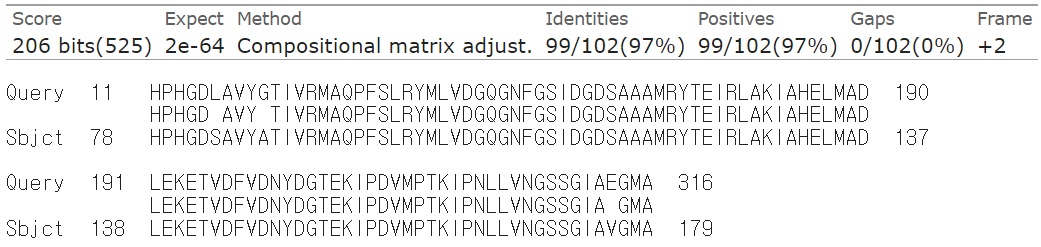


**Figure S1-28.** LC716502 (SSC-34) blast alignment.


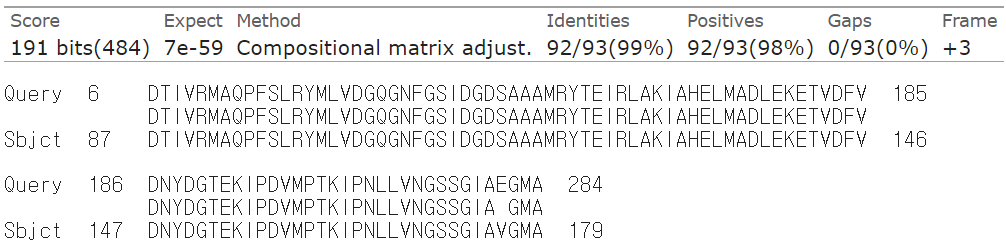


**Figure S1-29.** LC716503 (SSC-35) blast alignment.


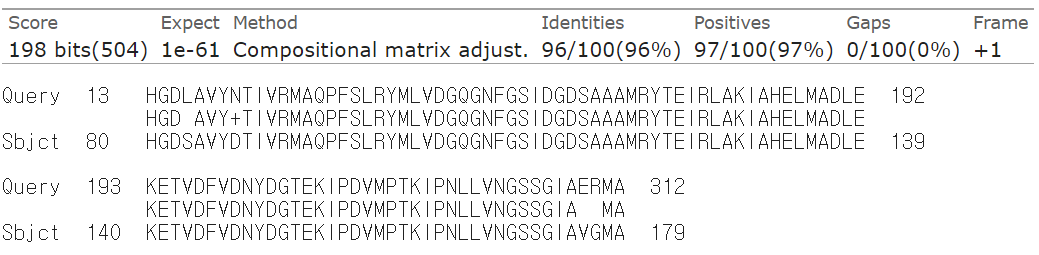


**Figure S1-30.** LC716504 (SSC-36) blast alignment.


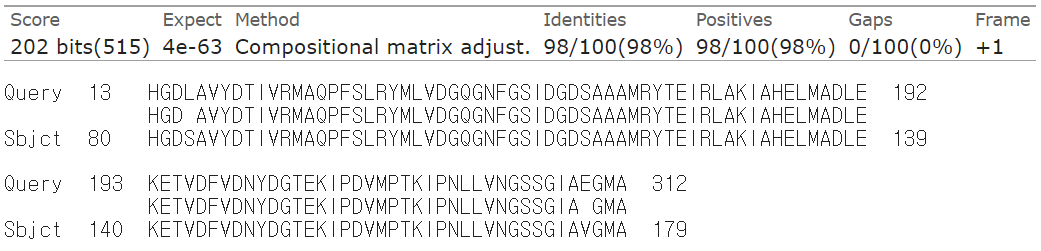


**Figure S1-31.** LC716505 (SSC-37) blast alignment.


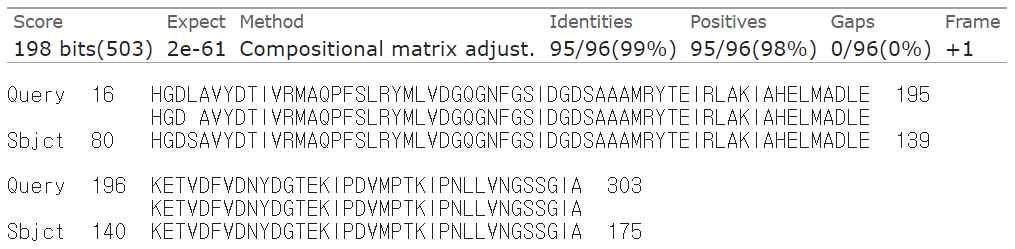


**Figure S1-32.** LC716506 (SSC-38) blast alignment.


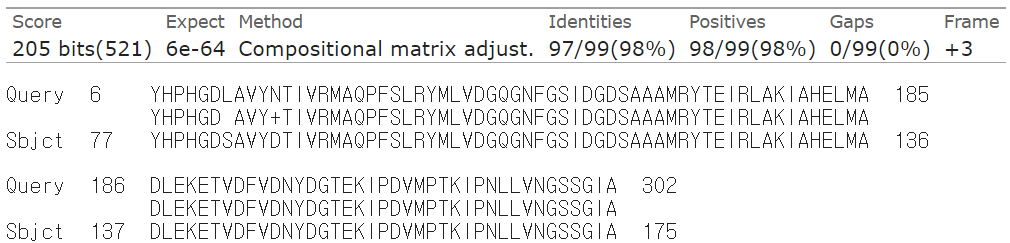


**Figure S1-33.** LC716507 (SSC-39) blast alignment.


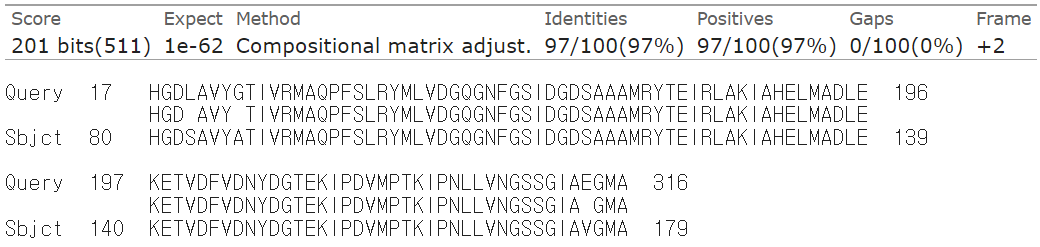


**Figure S1-34.** LC716508 (SSC-40) blast alignment.


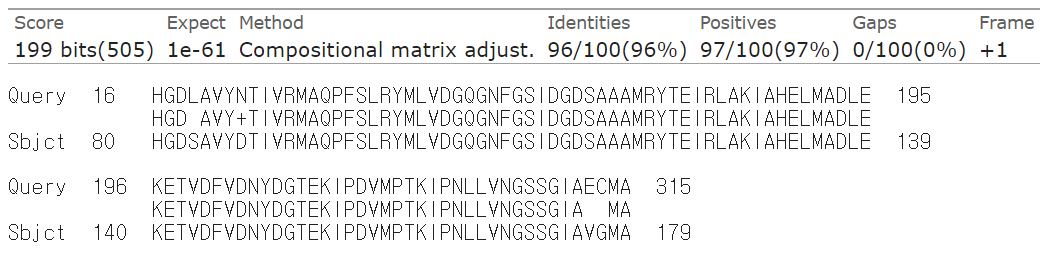


**Figure S1-35.** LC716509 (SSC-41) blast alignment.


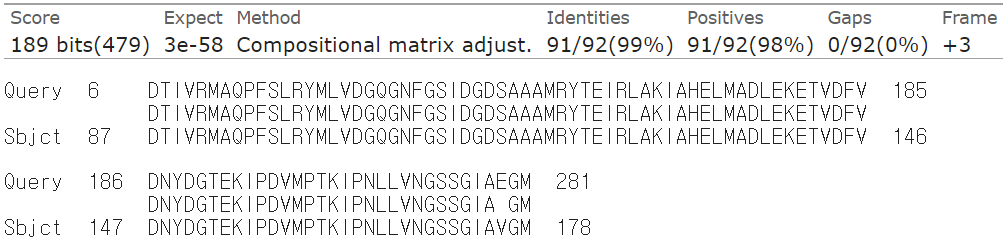


**Figure S1-36.** LC716510 (SSC-42) blast alignment.


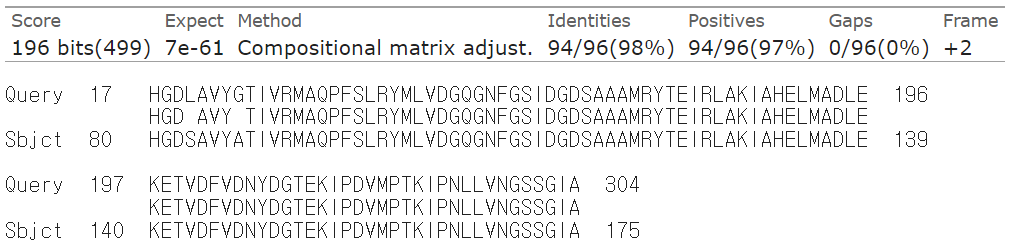


**Figure S1-37.** LC716511 (SSC-43) blast alignment.


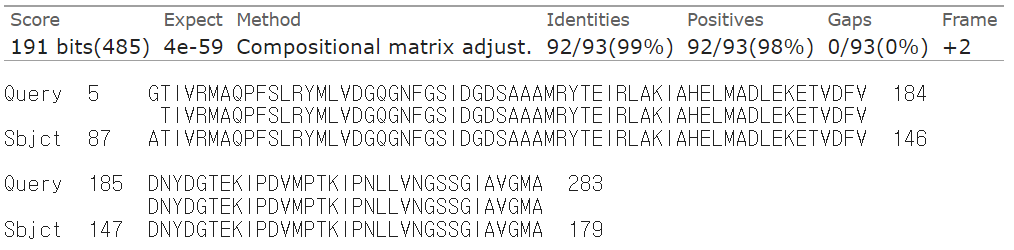


**Figure S1-38.** LC716512 (SSC-44) blast alignment.


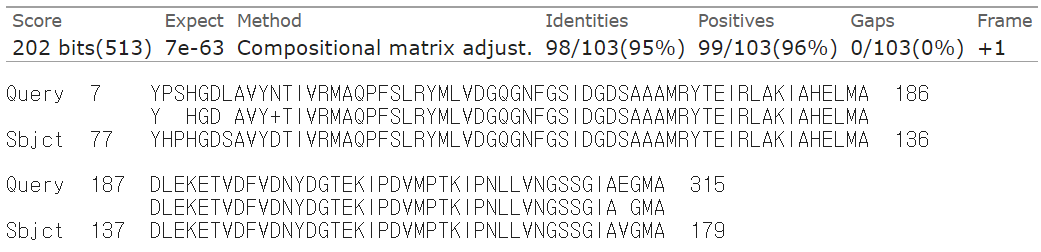


**Figure S1-39.** LC716513 (SSC-45) blast alignment.


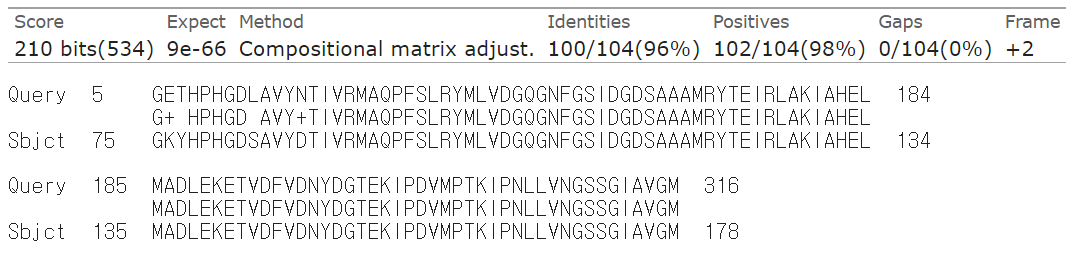


**Figure S1-40.** LC716514 (SSC-46) blast alignment.


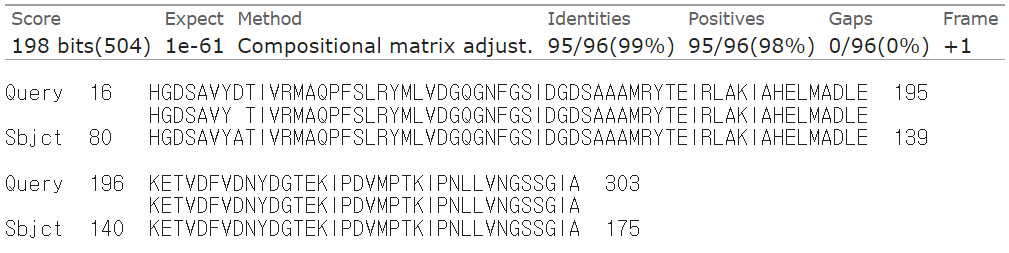


**Figure S1-41.** LC716515 (SSC-47) blast alignment.


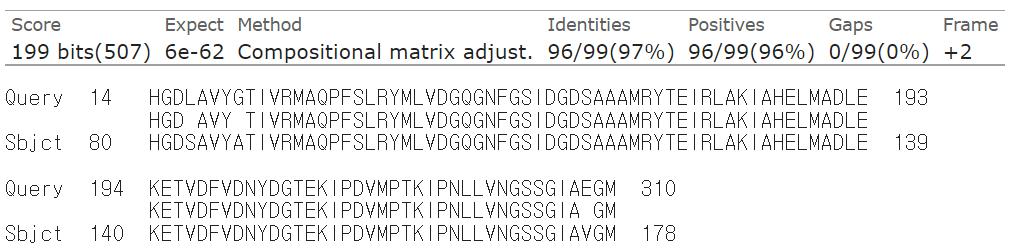


**Figure S1-42.** LC716516 (SSC-48) blast alignment.


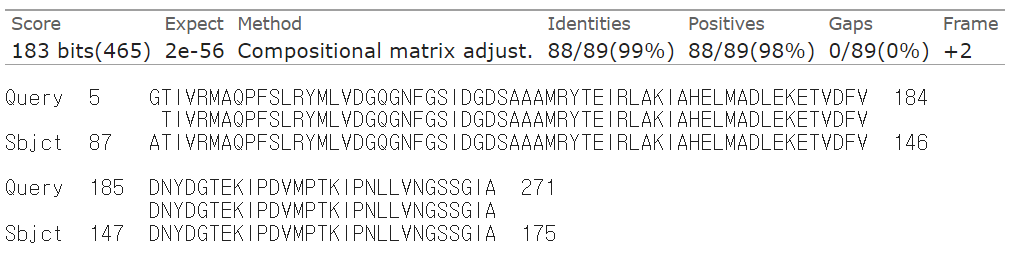


**Figure S1-43.** LC716517 (SSC-49) blast alignment.
