# Supplementary material for: Molecular characteristics of fluoroquinolone-resistant Escherichia coli isolated from suckling piglets with colibacillosis
Source: BMC Microbiol. 2022 Sep 15;22:216. doi: 10.1186/s12866-022-02632-9 (PMC9476276; doi:10.1186/s12866-022-02632-9)
Supplement: Supplementary file 2 — Additional file 2: Table S1. Primers used for PCR and DNA sequencing. [file 12866_2022_2632_MOESM2_ESM.docx]

**Table S1.** Primers used for PCR and DNA sequencing

| Group | Target | Sequence (5’→3’) | Size (bp) | Reference |
| --- | --- | --- | --- | --- |
| PMQR genes | *qnrA* | TCAGCAAGAGGATTTCTCA | 627 | [20] |
|  |  | GGCAGCACTATTACTCCCA |  |  |
|  | *qnrB* | CGACCTGAGCGGCACTGAAT | 515 | [21] |
|  |  | TGAGCAACGATGCCTGGTAG |  |  |
|  | *qnrC* | GGGTTGTACATTTATTGAATC | 447 | [22] |
|  |  | TCCACTTTACGAGGTTCT |  |  |
|  | *qnrD* | CGAGATCAATTTACGGGGAATA | 582 | [23] |
|  |  | AACAAGCTGAAGCGCCTG |  |  |
|  | *qnrS* | ACCTTCACCGCTTGCACATT | 571 | [21] |
|  |  | CCAGTGCTTCGAGAATCAGT |  |  |
|  | *aac(6’)-Ib-cr* | TTGCGATGCTCTATGAGTGGCTA | 482 | [10] |
|  |  | CTCGAATGCCTGGCGTGTTT |  |  |
|  | *qepA* | CGTGTTGCTGGAGTTCTTC | 403 | [24] |
|  |  | CTGCAGGTACTGCGTCATG |  |  |
| Aminoglycoside-modifying enzymes | *aac(3)-II* | TGAAACGCTGACGGAGCCTC | 369 | [25] |
|  |  | GTCGAACAGGTAGCACTGAG |  |  |
|  | *ant(2’’)-I* | GGGCGCGTCATGGAGGAGTT | 740 | [25] |
|  |  | TATCGCGACCTGAAAGCGGC |  |  |
| B-lactamases | TEM | CATTTCCGTGTCGCCCTTATTC | 800 | [26] |
|  |  | CGTTCATCCATAGTTGCCTGAC |  |  |
|  | SHV | AGCCGCTTGAGCAAATTAAAC | 713 | [26] |
|  |  | ATCCCGCAGATAAATCACCAC |  |  |
|  | OXA | GGCACCAGATTCAACTTTCAAG | 564 | [26] |
|  |  | GACCCCAAGTTTCCTGTAAGTG |  |  |
|  | CTX-M group 1 | TTAGGAARTGTGCCGCTGYA | 688 | [26] |
|  |  | CGATATCGTTGGTGGTRCCAT |  |  |
|  | CTX-M group 2 | CGTTAACGGCACGATGAC | 404 | [26] |
|  |  | CGATATCGTTGGTGGTRCCAT |  |  |
|  | CTX-M group 9 | TCAAGCCTGCCGATCTGGT | 561 | [26] |
|  |  | TGATTCTCGCCGCTGAAG |  |  |
|  | CTX-M group 8/25 | AACRCRCAGACGCTCTAC | 326 | [26] |
|  |  | TCGAGCCGGAASGTGTYAT |  |  |
|  | ACC | CACCTCCAGCGACTTGTTAC | 346 | [26] |
|  |  | GTTAGCCAGCATCACGATCC |  |  |
|  | FOX | CTACAGTGCGGGTGGTTT | 162 | [26] |
|  |  | CTATTTGCGGCCAGGTGA |  |  |
|  | MOX | GCAACAACGACAATCCATCCT | 895 | [26] |
|  |  | GGGATAGGCGTAACTCTCCCAA |  |  |
|  | CIT | CGAAGAGGCAATGACCAGAC | 538 | [26] |
|  |  | ACGGACAGGGTTAGGATAGY |  |  |
|  | DHA | TGATGGCACAGCAGGATATTC | 997 | [26] |
|  |  | GCTTTGACTCTTTCGGTATTCG |  |  |
|  | EBC | CGGTAAAGCCGATGTTGCG | 683 | [26] |
|  |  | AGCCTAACCCCTGATACA |  |  |
|  | GES | AGTCGGCTAGACCGGAAAG | 399 | [26] |
|  |  | TTTGTCCGTGCTCAGGAT |  |  |
|  | PER | GCTCCGATAATGAAAGCGT | 520 | [26] |
|  |  | TTCGGCTTGACTCGGCTGA |  |  |
|  | VEB | CATTTCCCGATGCAAAGCGT | 648 | [26] |
|  |  | CGAAGTTTCTTTGGACTCTG |  |  |
| Chloramphenicol | *catA1* | AGTTGCTCAATGTACCTATAACC | 547 | [27] |
|  |  | TTGTAATTCATTAAGCATTCTGCC |  |  |
|  | *cmlA* | CCGCCACGGTGTTGTTGTTATC | 698 | [27] |
|  |  | CACCTTGCCTGCCCATCATTAG |  |  |
| Sulfonamide | *sul1* | CTTCGATGAGAGCCGGCGGC | 433 | [28] |
|  |  | GCAAGGCGGAAACCCGCGCC |  |  |
|  | *sul2* | CGGCATCGTCAACATAACC | 722 | [29] |
|  |  | GTGTGCGGATGAAGTCAG |  |  |
| Tetracyclines | *tetA* | GTAATTCTGAGCACTGTCGC | 956 | [30] |
|  |  | CTGCCTGGACAACATTGCTT |  |  |
|  | *tetB* | CTCAGTATTCCAAGCCTTTG | 414 | [30] |
|  |  | ACTCCCCTGAGCTTGAGGGG |  |  |
|  | *tetC* | CCTCTTGCGGGATATCGTCC | 505 | [30] |
|  |  | GGTTGAAGGCTCTCAAGGGC |  |  |
|  | *tetD* | GGATATCTCACCGCATCTGC | 436 | [30] |
|  |  | CATCCATCCGGAAGTGATAGC |  |  |
|  | *tetE* | AAACCACATCCTCCATACGC | 278 | [30] |
|  |  | AAATAGGCCACAACCGTCAG |  |  |
|  | *tetG* | GCTCGGTGGTATCTCTGCTC | 468 | [30] |
|  |  | AGCAACAGAATCGGGAACAC |  |  |
| Integrons and cassettes | Class 1 integron | GCCTTGCTGTTCTTCTACGG | 558 | [31] |
|  |  | GATGCCTGCTTGTTCTACGG |  |  |
|  | Class 1 cassettes | GGCATCCAAGCAGCAAG | variable | [31] |
|  |  | AAGCAGACTTGACCTGA |  |  |
|  | Class 2 integron | CACGGATATGCGACAAAAAGGT | 788 | [32] |
|  |  | GTAGCAAACGAGTGACGAAATG |  |  |
|  | Class 2 cassettes | CGGGATCCCGGACGGCATGCACGATTTGT | variable | [32] |
|  |  | GATGCCATCGCAAGTACGAG |  |  |

**References**

20. Wang M, Tran JH, Jacoby GA, Zhang Y, Wang F, Hooper DC. Plasmid-mediated quinolone resistance in clinical isolates of *Escherichia coli* from Shanghai, China. Antimicrob Agents Chemother 2003;47:2242–8.

21. Jiang Y, Zhou Z, Qian Y, Wei Z, Yu Y, Hu S, et al. Plasmid-mediated quinolone resistance determinants *qnr* and *aac(6’)-Ib-cr* in extended-spectrum beta-lactamase-producing *Escherichia coli* and *Klebsiella pneumoniae* in China. J Antimicrob Chemother 2008;61:1003–6.

22. Wang M, Guo Q, Xu X, Wang X, Ye X, Wu S, et al. New plasmid-mediated quinolone resistance gene, *qnrC*, found in a clinical isolate of proteus mirabilis. Antimicrob Agents Chemother 2009;53:1892–7.

23. Cavaco LM, Hasman H, Xia S, Aarestrup FM. *qnrD*, a novel gene conferring transferable quinolone resistance in Salmonella enterica serovar Kentucky and Bovismorbificans strains of human origin. Antimicrob Agents Chemother 2009;53:603–8.

24. Yamane K, Wachino J, Suzuki S, Kimura K, Shibata N, Kato H, et al. New plasmid-mediated fluoroquinolone efflux pump, *QepA*, found in an *Escherichia coli* clinical isolate. Antimicrob Agents Chemother 2007;51:3354–60.

25. Sandvang D, Aarestrup FM. Characterization of aminoglycoside resistance genes and class 1 integrons in porcine and bovine gentamicin-resistant *Escherichia coli*. Microb Drug Resist 2000;6:19–27.

26. Dallenne C, Da Costa A, Decré D, Favier C, Arlet G. Development of a set of multiplex PCR assays for the detection of genes encoding important beta-lactamases in Enterobacteriaceae. J Antimicrob Chemother 2010;65 490–5.

27. Van TT, Chin J, Chapman T, Tran LT, Coloe PJ. Safety of raw meat and shellfish in Vietnam: an analysis of *Escherichia coli* isolations for antibiotic resistance and virulence genes. Int J

Food Microbiol 2008;124:217–23.

28. Sandvang D, Aarestrup FM, Jensen LB. Characterisation of integrons and antibiotic resistance genes in Danish multiresistant *Salmonella* *enterica* Typhimurium DT104. FEMS Microbiol Lett 1998;160:37–41.

29. Maynard C, Fairbrother JM, Bekal S, Sanschagrin F, Levesque RC, Brousseau R, et al. Antimicrobial resistance genes in enterotoxigenic *Escherichia coli* O149:K91 isolates obtained over a 23-year period from pigs. Antimicrob Agents Chemother 2003;47:3214–21.

30. Sengeløv G, Agersø Y, Halling-Sørensen B, Baloda SB, Andersen JS, Jensen LB. Bacterial antibiotic resistance levels in Danish farmland as a result of treatment with pig manure slurry. Environ Int 2003;28:587–95.

31. Ng LK, Mulvey MR, Martin I, Peters GA, Johnson W. Genetic characterization of antimicrobial resistance in Canadian isolates of *Salmonella Serovar* Typhimurium DT104. Antimicrob Agents Chemother 1999;43:3018–21.

32. Sáenz Y, Briñas L, Domínguez E, Ruiz J, Zarazaga M, Vila J, et al. Mechanisms of resistance in multiple-antibioticresistant *Escherichia coli* strains of human, animal, and food Origins. Antimicrob Agents Chemother 2004;48:3996–4001.
